# Mapping regional livelihood benefits from local ecosystem services assessments in rural Sahel

**DOI:** 10.1371/journal.pone.0192019

**Published:** 2018-02-01

**Authors:** Katja Malmborg, Hanna Sinare, Elin Enfors Kautsky, Issa Ouedraogo, Line J. Gordon

**Affiliations:** 1 Stockholm Resilience Centre, Stockholm University, Stockholm, Sweden; 2 CGIAR Program on Climate Change, Agriculture and Food Security (CCAFS), ICRASAT West & Central Africa Regional Office, Bamako, Mali; Bristol University/Remote Sensing Solutions Inc., UNITED STATES

## Abstract

Most current approaches to landscape scale ecosystem service assessments rely on detailed secondary data. This type of data is seldom available in regions with high levels of poverty and strong local dependence on provisioning ecosystem services for livelihoods. We develop a method to extrapolate results from a previously published village scale ecosystem services assessment to a higher administrative level, relevant for land use decision making. The method combines remote sensing (using a hybrid classification method) and interviews with community members. The resulting landscape scale maps show the spatial distribution of five different livelihood benefits (nutritional diversity, income, insurance/saving, material assets and energy, and crops for consumption) that illustrate the strong multifunctionality of the Sahelian landscapes. The maps highlight the importance of a diverse set of sub-units of the landscape in supporting Sahelian livelihoods. We see a large potential in using the resulting type of livelihood benefit maps for guiding future land use decisions in the Sahel.

## Introduction

The ecosystem services concept is widely used to illustrate the many ways people depend on ecosystem processes for their well-being. Ecosystem services are defined as the benefits that people obtain from ecosystems, and can be separated into four general categories: supporting, provisioning, regulating and cultural services [1]. The direct dependency on provisioning ecosystem services for livelihoods is particularly high in rural regions with widespread poverty. Therefore, understanding the distribution of ecosystem services in rural landscapes can help inform decisions about investments in poverty alleviation [2,3]. There has been an explosion of literature on spatial mapping of ecosystem services, however several reviews in this field have shown that mapping studies mainly use secondary data such as land cover maps and national or global databases rather than field data [4–8].

In poor rural regions, smallholder farming and pastoralism are dominant land uses [9,10], which means that these landscapes are highly managed by humans. This shows the importance of an ecosystem services perspective that recognizes both the social and the ecological processes involved in ecosystem service generation [11]. However, current methods to map ecosystem services either rely on ecological indicators only [8], or the methods require substantial amounts of input data to represent both social and biophysical aspects (see e.g. [12,13] for some recent examples). Such data is seldom available for regions with widespread poverty, so new approaches to mapping and assessing ecosystem services relevant for local populations in these landscapes are needed [14,15]. Such approaches need to take local knowledge and needs into account at the same time as being generalizable to the scale and resolution relevant for informing decision making and development interventions [6]. Most recent approaches to mapping ecosystem services that have tried to bridge the data gap in regions with widespread poverty have either been local in scale [14,16,17] or have mainly been based on expert assessments rather than local knowledge [15].

Remote sensing has the potential to bridge the gap between local level data collection, and larger-scale classifications. While remote sensing sometimes has been used for ecosystem services mapping [18,19], a problem has been that most of the no-cost earth observation data available have coarse resolution. This means that it is not suitable for object-based image analyses where data can be used to capture both biophysical and social processes [20–22]. A method using solely remotely sensed low to mid-resolution data to assess ecosystem service provision thus runs the risk of losing the detail needed to understand the local user perspective.

In this study we build on a detailed community based ecosystem services assessment that was conducted in six village landscapes in Burkina Faso in 2011–2012 [23] and investigate the potential to extrapolate these results to a more policy-relevant scale. In the former study, Sinare and colleagues [23] identified a set of social-ecological patches (“landscape units that correspond with the words that local people use when describing their landscapes, characterized by a combination of land use, land cover and topography”) across these landscapes, as well as the ecosystem services and livelihood benefits associated with these social-ecological patches. Here, we develop a way to extrapolate the data-intense community based method, using participatory transect walks, remotely sensed imagery and a hybrid classification method, to a scale at which decisions about larger investments in the landscape are often taken. Based on the resulting maps, we analyze the spatial distribution across this study area of five different livelihood benefits (annual crops for consumption; nutritional diversity and medicinal uses; material assets and energy; saving/insurance; and income) that are derived from the provisioning ecosystem services. Finally, we discuss what the resulting livelihood benefit maps show us about the landscape and how the maps could be used.

## Background: Ecosystem service dependence in village landscapes in the Sahel

Approximately 90 percent of the population in the Sudano-Sahelian zone of Burkina Faso lives in rural areas [24] and have agriculture as the main livelihood strategy [25]. More than 44 percent live below the poverty line (US$ 1.25 a day), and in 2010 a majority of these people suffered from multidimensional poverty [26]. Livestock keeping is an integrated part of agricultural practices [27], and gathering of wild plants for food, medicine, construction material and spiritual uses constitutes an important additional land use [28,29]. People are therefore highly integrated in their local landscapes and depend on a range of local ecosystem services, which provide them with livelihood benefits such as nutrition, income and insurance. They are thus also greatly affected by the variable landscape productivity following the low and highly variable rainfall in the region.

Rainfall in the Sudano-Sahelian zone of Burkina Faso ranges from 500 to 900 mm yr^-1^ on average in a north-south gradient [30] with high inter-annual variation in total amount and distribution over the season. Temperature and rainfall variability is expected to increase due to climate change [31]. Due to the combination of widespread poverty, high reliance on agriculture and the expected changes in climate, countries in the Sudano-Sahelian region are targeted in many government and NGO initiatives for poverty alleviation and natural resources management. The official development assistance to agriculture in Burkina Faso, Mali, Niger and Senegal was US$ 620 million in 2010 [32]. These investments are divided across activities such as the promotion of soil and water conservation techniques [33]. Another project is the Great Green Wall for the Sahara and Sahel initiative. These agricultural development initiatives mostly focused on tree planting and sustainable land management practices [34], the development of small-scale irrigation schemes [35], and generally aim to promote specific provisioning ecosystem services, such as crops and wood production. However, they seldom address multiple ecosystem services simultaneously. Tools that can help assess the trade-offs and synergies between different ecosystem services that might arise from these interventions [36], and the consequences for livelihoods, would be useful to guide future development investments in the region.

**Fig 1:**
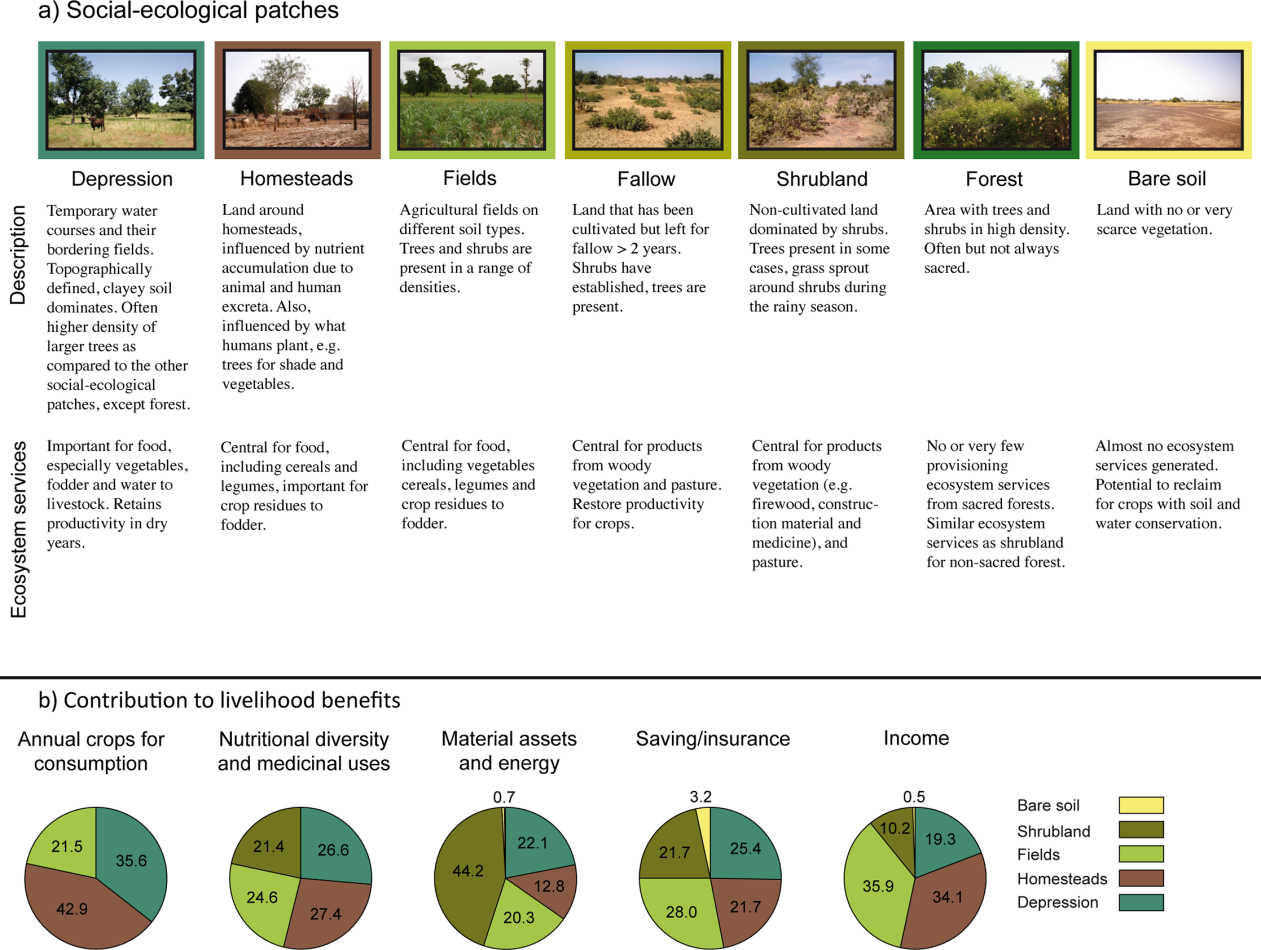
Description of social-ecological patches and their contribution to livelihood benefits. a) Social-ecological patches and characteristics of their ecosystem services generation; b) Relative contribution of the social-ecological patches found in the current study to the five identified livelihood benefits. Original scores are means from 36 focus groups conducted by Sinare and colleagues [23]. Scores here are adapted from the original work, based on feedback during fieldwork conducted for this paper, to better represent per unit area contribution of social-ecological patches (for detailed description, see S1 Text). Figure partly based on Sinare and colleagues [23] under a CC BY license, with permission from the authors, original copyright 2016.

In a previous study, Sinare and colleagues [23] developed a method for identifying multiple provisioning ecosystem services in village landscapes in Burkina Faso. Sinare and colleagues [23] used a multi-method approach, which included participatory mapping, transect walks and focus group discussions with local villagers in six different villages, to identify key social-ecological patches, provisioning ecosystem services generated from these, and livelihood benefits derived from the ecosystem services (Fig 1). The social-ecological patches identified through participatory mapping and transect walks are depression, homesteads, fields, fallow, shrubland, forest and bare soil. The separation between ecosystem services and benefits followed de Groot and colleagues [37], where ecosystem services are defined as ecosystem functions with the potential to directly and indirectly satisfy different aspects of human well-being, while the actual use of ecosystem services provides benefits. The five identified livelihood benefits are annual crops for consumption, nutritional diversity and medicinal uses, material assets and energy, savings/insurance, and income. The attribution of a value for each livelihood benefit to each social-ecological patch was based on scoring by villagers in 36 different focus groups (three with women and three with men in each of the six villages). Using transect points and visual interpretation of high-resolution remotely sensed imagery, the cover and spatial distribution of the social-ecological patches in the six villages could then be mapped. For detailed methods, see Sinare and colleagues [23]. See S1 Fig for schematic illustrating the steps of the method in Sinare and colleagues [23] and in the current study.

## Materials and methods

### Defining the scale of analysis

**Fig 2:**
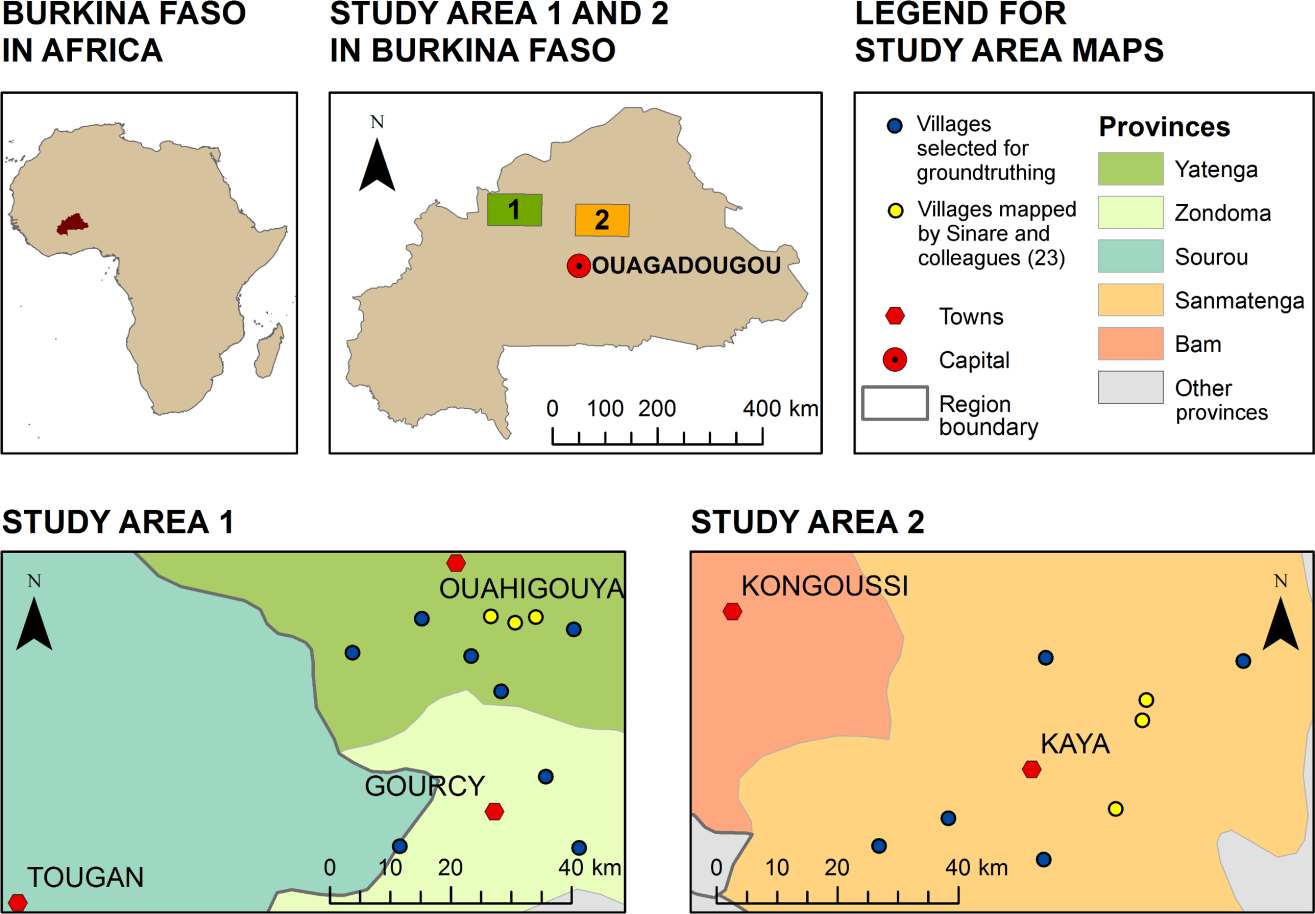
Overview maps. Maps of the study areas and villages where fieldwork was conducted. Study areas indicate extent of analyzed remote sensing images. Points show location of villages included in this study (blue) and mapped by Sinare and colleagues [23] (yellow). Boundary data is from Natural Earth [41] and location of villages and towns collected with GPS during fieldwork.

Burkina Faso is administratively organized into 13 larger regions, which are further divided into 45 provinces, 351 municipalities and more than 8000 villages. This study focuses on the provincial scale within the regions Nord and Centre-Nord, located in the semi-arid Sudano-Sahelian agro-ecological zone in northern Burkina Faso (Fig 2). The study includes fieldwork in the two regions, within which all six villages mapped by Sinare and colleagues [23] are located. The mean size of a province in Burkina Faso is 6000 square kilometers, which is why we chose this as the size for our study areas. The position of the study areas was adjusted to be covered by a single Landsat scene each, to avoid problems with merging images captured at different times and atmospheric conditions. Study area 1 covers the Yatenga, Zondoma and Sourou provinces while study area 2 covers the Sanmatenga and Bam provinces. The provinces have a similar biophysical and cultural context: The landscape in the studied provinces mainly consists of mosaic vegetation/croplands, rainfed croplands or sparse vegetation, with small areas of open grassland [38], and the composition of the population is 88–90% Mooré speaking and 6–9% Fulfuldé speaking [39,40]. Once the study areas were defined, eight villages were chosen in area 1 and five villages in area 2 (Fig 2).

The number of villages in each study area was kept low due to logistical constraints. However, the small sample size was partly balanced by conducting several transect walks in each village, meaning that the within-village landscape variation was covered by the collected data. Selection of villages was also done so as to increase the chances of covering between-village variation by applying a combination of criteria: First, the villages were selected to include both larger and smaller villages, in terms of population, than the regional average sized villages that were studied by Sinare and colleagues [23], but not big enough to be classified as towns. Population in the selected villages ranged from 600 to 2,900 inhabitants in the 2006 census [42]. The villages were also chosen based on their position within the study area, so that they were evenly spread out and covered areas that seemed to vary in land cover based on Normalized Difference Vegetation Index (NDVI) calculated from recent Landsat scenes. Lastly, the villages had to be accessible by car for practical reasons related to fieldwork logistics. We collaboratively chose study sites together with our local project partner Institut de l’Environnement et de Recherches Agricoles (INERA), a governmental research institute in Burkina Faso. Local authorities in the regional capitals Ouahigouya and Kaya were informed upon our arrival, and permission to work in individual communities was obtained from village leaders.

### Generalizability of social-ecological patches and ecosystems services

A participatory approach with transect walks and focus group discussions was used to investigate the generalizability of the social-ecological patches and connected sets of provisioning ecosystem services identified by Sinare and colleagues [23]. The fieldwork was conducted between October and December 2014. An elected community representative in each village was asked to select male and female respondents knowledgeable about the landscape, farming practices and land use history. To increase the likelihood to cover more social-ecological patches, the respondent groups were asked to choose a direction for the walk that would show the variety of the landscape in their village. Each walk was between 3–5 kilometers long, depending on the village size and the terrain, and kept as straight of a transect as possible. Two or three transects were conducted in each village and two to four villagers took part in each walk. During the walks, stops were made every 100 meters to record GPS coordinates, take photographs and respondents were asked to describe the activities and services extracted there. The previously defined social-ecological patches were not mentioned to the respondents in advance, allowing them to freely describe the landscape. All interviews and other communication with the villagers were conducted through a translator. In total, 27 transect walks were made and 784 GPS points gathered for calibration and groundtruthing of the maps.

Interview notes were coded in order to assign a social-ecological patch name to each transect point. To a large extent, villagers had used the same terminology and definitions as in the study by Sinare and colleagues [23]. For each village, the notes for each point were also summarized to connect provisioning ecosystem services with each social-ecological patch category. This method could not produce the same detailed score values as by Sinare and colleagues [23], but only verify if a service was present or absent in a social-ecological patch. Basic maps of the transects and identified social-ecological patches (including points that had not been possible to assign to any specific category) were produced using Google Earth, and were shown to the participants during focus group interviews in the villages. The ecosystem services provided by each social-ecological patch as identified in the study by Sinare and colleagues [23] were presented together with pictures of the social-ecological patches. Questions were asked regarding clarifications or observations from the transect walks, and the participants were given the chance to comment and criticize the maps or definitions of the social-ecological patches. Participants were also asked to rank social-ecological patches in order of importance (high/low) for the supply of different services, allowing more nuanced data on the connection between an ecosystem service and a social-ecological patch than achieved during the transect walks.

### Development of a hybrid classification method

**Fig 3:**
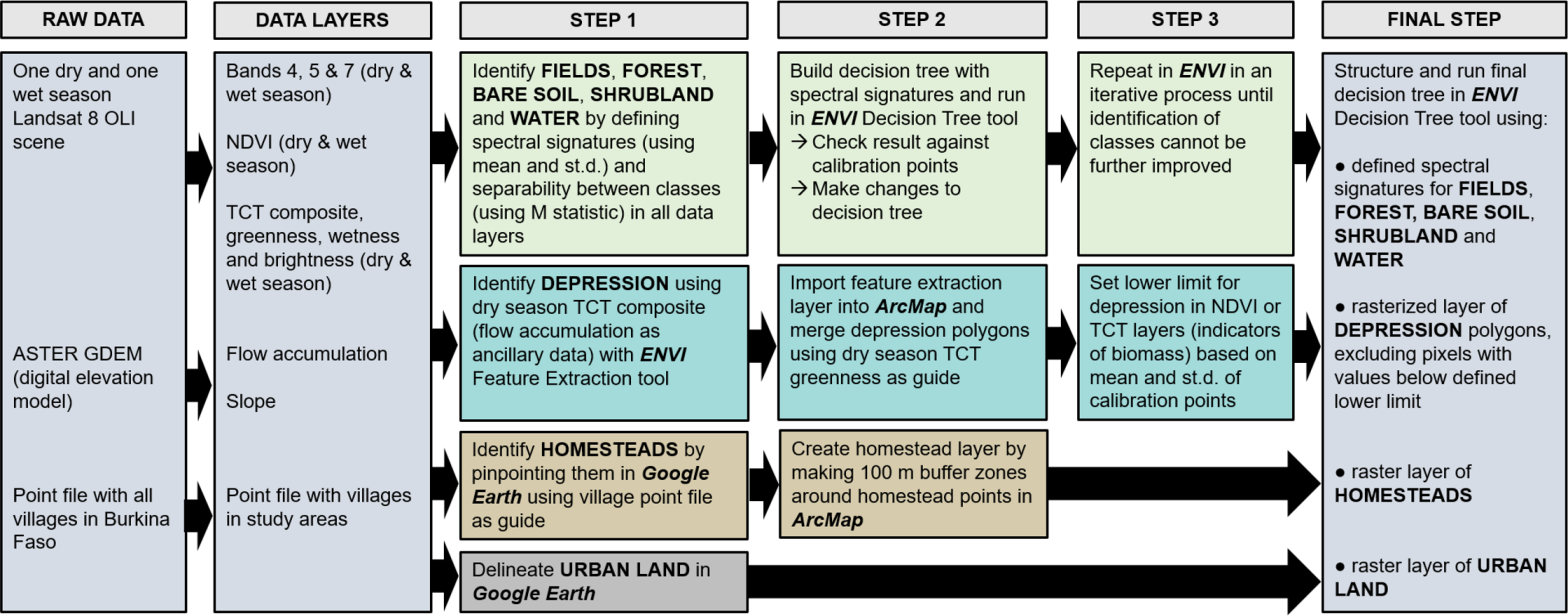
Schematic of work flow for the developed hybrid classification method. The data layers used include the red, near infrared and mid-infrared bands of the Landsat 8 OLI scenes (bands 4, 5 and 7); Normalized difference vegetation index (NDVI; [52,53]); and Tasseled Cap Transformation (TCT), an index that compresses multispectral Landsat data into the three bands brightness, greenness and wetness [54,55]. To define the spectral signatures of some of the social-ecological patches, we used mean and standard deviations (st.d.) as well as the M statistic (a measure of spectral separability between classes; [56,57]) for the different patch calibration points in all data layers. For implementation of the method, three GIS softwares were used: ArcMap [58], ENVI [43] and Google Earth.

The classification method was structured in a decision tree using one dry season and one rainy season Landsat 8 OLI scene, the Advanced Spaceborne Thermal Emission and Reflection Radiometer (ASTER) global digital elevation model (GDEM), and calibration data from fieldwork and previous village maps [23]. Landsat 8 OLI was an appropriate sensor for this scale of study because at the time of analysis it was the highest resolution open access imagery available. A decision tree was developed for classification of the data using the decision tree classifier toolkit in the ENVI software [43]. Decision tree classifiers are a type of supervised classification that divides the data into two groups in a binary fashion at every node based on defined criteria for one or several data layers, where the end of every branch is one class [44,45]. A decision tree classifier is useful as it allows for several data layers (Table 1), making it possible to include ancillary data such as digital elevation models and vegetation indices. This has proven to increase accuracy in semi-arid landscapes where spectral signatures from bare soil, sparse vegetation and urban areas tend to be similar, making them hard to separate from each other [46]. It also allows for hybridization, combining other types of classification methods as preparatory steps in some of the data layers [47]. Due to the patchiness of the landscape in the study areas and the relatively poor resolution of the remotely sensed data, a combination of spectral signatures, object-based image analysis [21], and visual interpretation was used to classify the different social-ecological patches in several preparatory steps, before the final decision tree could be constructed (Fig 3). For detailed description of the hybrid classification method and the final decision trees, see S2 Text or [48].

**Table 1:** Input data for maps of social-ecological patches.

Study area	Input data for maps of social-ecological patches
**1**	Landsat 8 OLI scene LC81960512014054LGN00; date February 23, 2014; dry season; 30 m resolution. [49]
	Landsat 8 OLI scene LC81960512014278LGN00; date October 5, 2014; rainy season; 30 m resolution. [49]
**2**	Landsat 8 OLI scene LC81950512014079LGN00; date March 20, 2014; dry season; 30 m resolution. [49]
	Landsat 8 OLI scene LC81950512014271LGN00; date September 28, 2014; rainy season; 30 m resolution. [49]
**Both**	ASTER GDEM V2 (digital elevation model; 30 m resolution), a product of METI and NASA. ASTER L1B: ASTGTM2_N13W003, ASTGTM2_N13W004, ASTGTM2_N12W001, ASTGTM2_N12W002, ASTGTM2_N13W001, ASTGTM2_N13W002. [50]
	Point file with all villages in Burkina Faso. Bf_loc.shp. [51]

### Conversion of social-ecological patch maps into livelihood benefit maps

Each social-ecological patch generates multiple ecosystem services, which in turn are associated with multiple livelihood benefits, albeit to different degrees. We used a scoring system to convert the social-ecological patch maps into benefit maps. The scores are adapted from the in-depth fieldwork conducted by Sinare and colleagues [23] and represent the relative contribution of each patch (per unit patch area) to the different livelihood benefits (see Fig 1B and S1 Text).

The amount of benefits from a social-ecological patch can depend on location and overall spatial extent. Focus group discussions, transect walks and validation workshops revealed that for fields and shrubland, all benefits except saving/insurance decrease in value the further they are situated from a homestead. This is due to the increasing effort to walk and transport goods from further afield that makes it harder for the villagers to benefit from these patches. To account for this, we created a weight raster based on 5 buffer zones around houses, where the zone closest to a house was given the weight 1.2, while the zone furthest away was assigned 0.8, based on rough translations of observed sizes of management rings in farming systems in northern Burkina Faso [59].

For shrubland, the benefit scores for nutritional diversity and medicinal uses, material assets and energy, and savings/insurance also depend on how much shrubland a village has (see Results). Shrubland patches can be too small or few in a village to generate the mentioned benefits well, while above an upper limit the added value of more shrubland decreases. To account for this, a weight raster was created for each village. First, village boundaries were made by creating Thiessen polygons in ArcMap [58] from the village location point file [51]. This is an approximation and does not represent the true boundaries of villages, but is considered a fair generalization when data on true village boundaries does not exist [60]. Second, the total amount of shrubland cover was calculated for each village from the social-ecological patch map, and these values in turn converted into five categories. Third, these categories were given weights between 0.8–1.2 based on the area-benefit relationship described above. The absolute weights for both homestead buffer zones and shrubland thresholds are our best estimation based on the qualitative assessment of data from focus group interviews and transect walks. The livelihood benefit maps were generated using the ArcMap Raster Calculator [58] to combine the social-ecological patch map with the livelihood benefit scores and assigned weights. For a summary of how the individual livelihood benefit maps were calculated, see S1 Table.

## Results

### Comparison of social-ecological patches and ecosystem services

**Fig 4:**
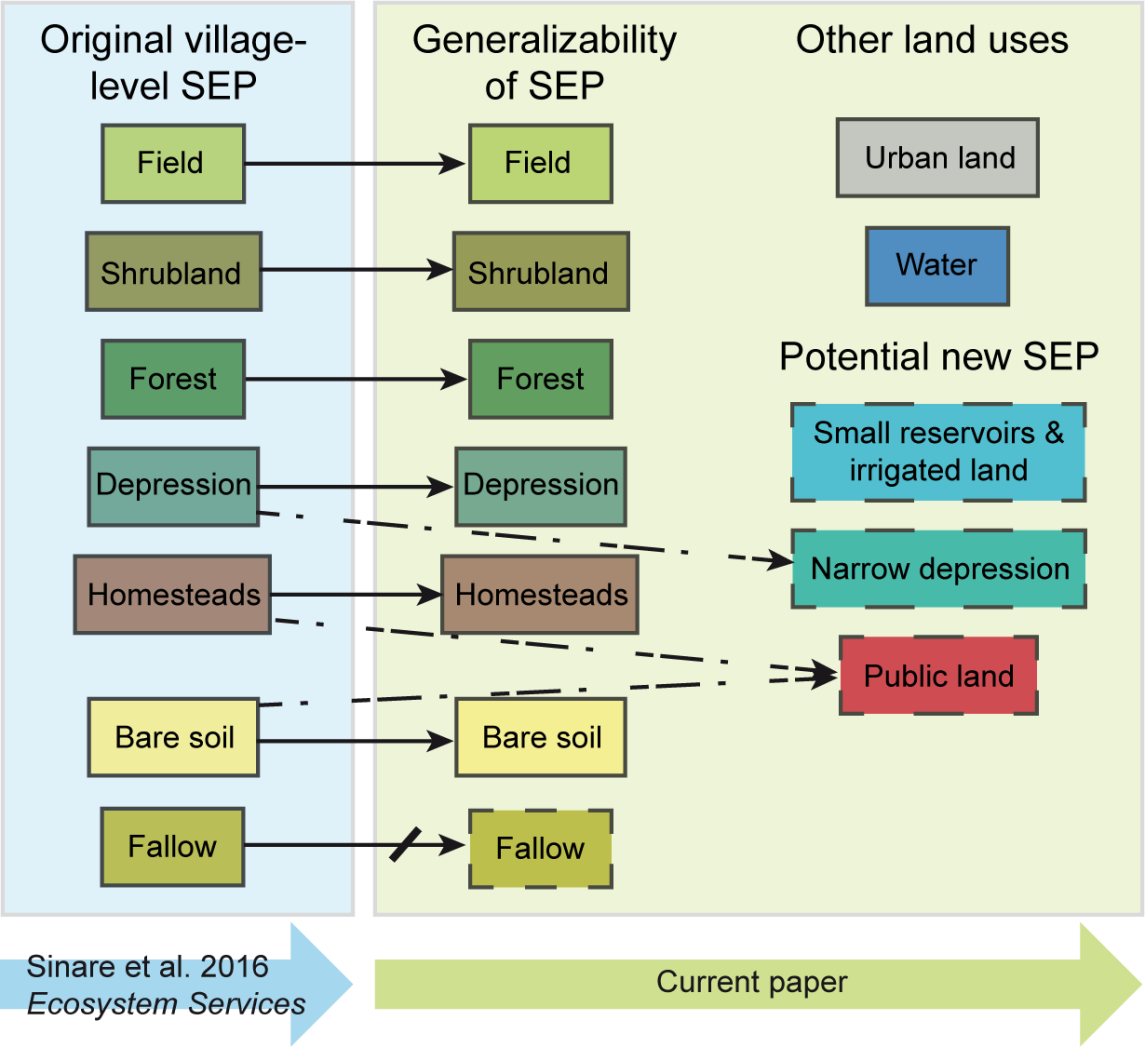
Schematic of the generalizability of the social-ecological patches. The dashed arrows indicate suggested new social-ecological patches that were not included by Sinare and colleagues [23]. The crossed arrow connected to fallow indicates the failure to extrapolate this patch using remotely sensed data (for explanation see Results). Social-ecological patches in dashed boxes were not mapped in the current study, either because they are additions to the original categorization or because it was impossible with the available data.

For the most part the social-ecological patches and the related sets of ecosystem services found in this study were the same as identified by Sinare and colleagues [23]. Fields and fallow were defined in the same way and generated the same sets of ecosystem services. However, some differences were observed as compared to the previous study (Fig 4). Shrublands provided the same set of ecosystem services everywhere, but depending on the landscape composition of the village, the services were valued differently between communities. In villages with barely any shrubland the value of this patch was very low as the area was too small to be used as pasture and to maintain the species diversity that would generate wild foods and medicine. In villages with little shrubland, the villagers tended to put more value on this social-ecological patch, even expressing that they had refrained from turning it into fields to maintain the ecosystem services (such as medicines and pasture) that shrublands provided. However, in villages with large areas of shrubland, the ecosystem services tended to be valued lower and the land seen more as remaining land that was not suitable for cultivation (see how we dealt with this in the mapping in Materials and methods). Most depressions also provided the same set of ecosystem services. However, in some of the villages, depressions were narrower and not arable, suggesting that there are two types of depressions. It was also suggested that small reservoirs and irrigated land could be classified as a social-ecological patch, since the access to irrigation, water for animals and aquaculture that they enable, produce a distinct set of provisioning ecosystem services not covered by the social-ecological patches identified by Sinare and colleagues [23]. In larger village centers, homesteads also covered areas of bare soil that were used as market places, for meetings and as football fields. These could potentially be defined as a new social-ecological patch called “public land”, as they provide a different set of cultural ecosystem services than bare soil, although their provisioning services are the same.

Although the level of detail in the data gathered during fieldwork for this study did not allow for a verification of the scoring values in the study by Sinare and colleagues [23], the general pattern of high or low importance of a patch for the supply of a specific ecosystem service corresponded with that of the previously collected scores. Therefore, our assumption is that the scoring values from Sinare and colleagues [23] would be applicable at this larger scale as well.

### Maps of social-ecological patches

**Fig 5:**
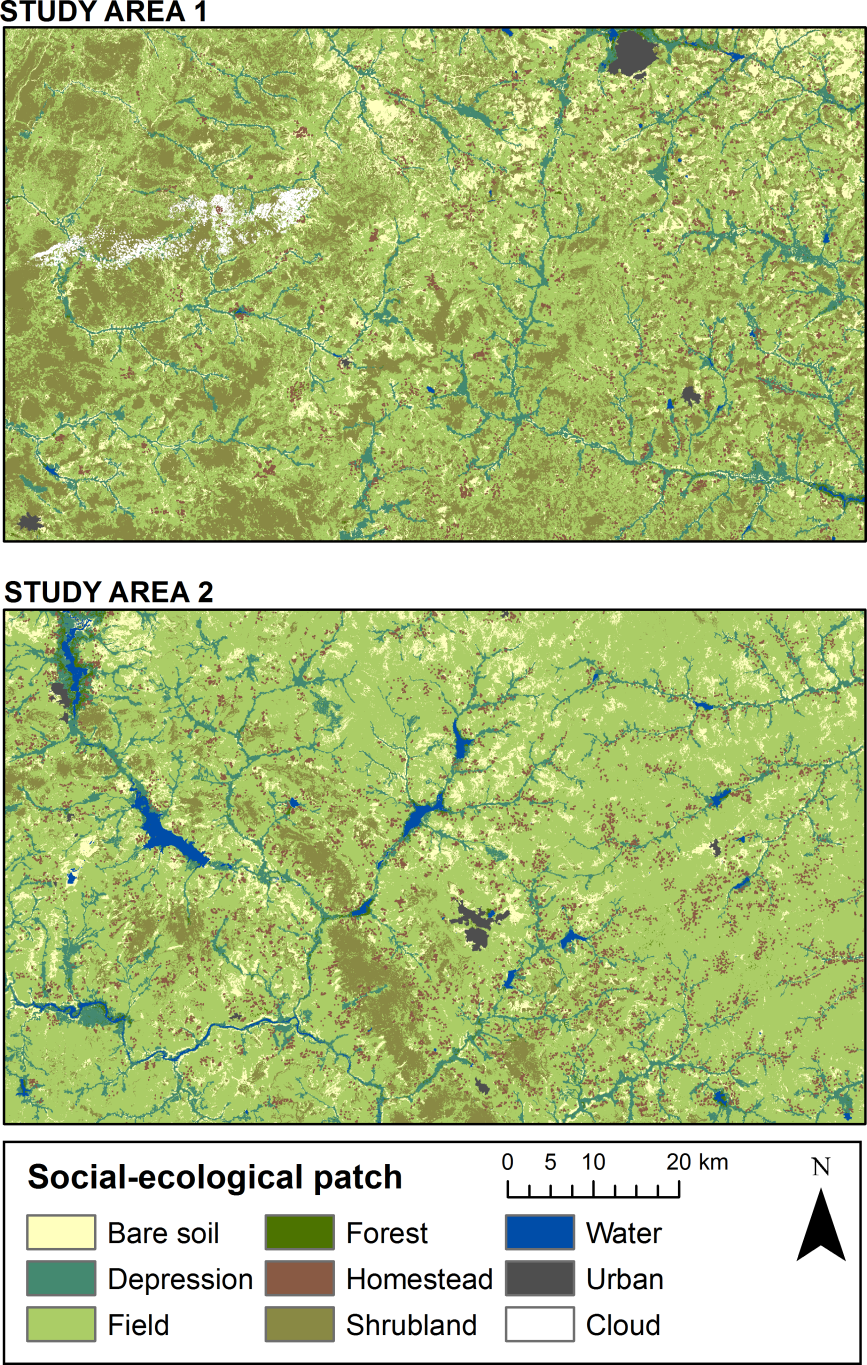
Maps of social-ecological patches for study area 1 and 2. Urban land and water is a land cover, but not a social-ecological patch.

The distribution patterns of social-ecological patches within the study areas is shown in the final maps produced using the hybrid classification method (Fig 5). In study area 1, the north-eastern corner appears to be drier, with a higher share of bare soil, while the south-eastern corner is wetter and has more depressions and water bodies. The western part of the study area is covered by more shrubland, which corresponds with this region being more topographically variable and less populated. In study area 2, the map indicates that the north-eastern part of the study area is quite homogenous, while the southern and western parts are variable, with water bodies, depressions and shrublands. The diagonal belt of shrublands that runs in the south-central part of the study area corresponds with a chain of small mountains and hills. Overall, the landscape is dominated by fields, covering 50–70 percent of the two study areas (Table 2). In study area 1, shrubland is also an important patch type, covering about 30 percent of the land surface. 10 percent of the land in both study areas was classified as bare soil.

**Table 2:** Percentage of landscape covered by each social-ecological patch and their relative accuracies^1^.

Social-ecological patch	Study area 1: Landcover (%)	Study area 1: Relative accuracy (%)	Study area 2: Landcover (%)	Study area 2: Relative accuracy (%)
Bare soil	10.9	90.0	7.7	96.0
Depression	6.0	83.3	7.5	87.2
Field	50.1	86.3	71.2	81.0
Forest	0.4	33.3	0.5	25.0
Homestead	2.8	91.5	5.2	95.2
Shrubland	29.1	50.0	6.4	11.4
*Water*	0.2	93.8	1.1	100
*Urban land*	0.5	-	0.4	-
**Overall accuracy**	-	74.4	-	77.8

^1^ Relative accuracy is an estimate of how well the maps capture the composition of the landscape, see Results.

To assess how well the maps capture the composition of the landscape, the accuracy was assessed using the following method: a 15 meter buffer was created around the groundtruthing points and the accuracy was judged based on if the buffer overlapped with a pixel assigned to the right class. This method adjusts for possible small location inaccuracies in the data and issues stemming from the satellite imagery being of relatively low resolution compared to the general size of the social-ecological patches. Instead of assessing the exactness of the maps, this method gives an indication of the relative accuracy of the classification (Table 2). For an assessment of the pixel-based exactness of the map, see a confusion matrix with producer’s and user’s accuracies in S2 Table.

Using the developed hybrid classification method, the relative accuracy is 81–96 percent for the social-ecological patches depression, homesteads, fields and bare soil. This indicates a good representation of the landscape composition as these social-ecological patches cover 70 percent of the total surface in study area 1 and 92 percent in study area 2. Shrubland and forest have relatively low accuracies. Many shrubland groundtruthing points are either classified as fields or as bare soil. This is because shrublands are mainly defined through indicators for biomass (see decision trees in S2 Text), meaning that when shrubs are sparse, that patch is often classified as bare soil in the maps due to its low biomass signature. However, due to the sparse vegetation, the generation of shrubland related ecosystem services is low in these places, meaning that this classification error has limited consequences for the assessment of the spatial distribution of the sets of ecosystem services. Forest patches in these landscapes are very rare, covering less than 1 percent of the land cover in the study by Sinare and colleagues [23]. Forests are also often sacred (for example, in the 13 villages visited for this study, half of the forest patches were sacred and either forbidden to enter or to harvest anything from). This means that the forest social-ecological patch is not as important for provisioning ecosystem services as other patches. Therefore, the low accuracy will not have a big impact on the result with regards to provisioning ecosystem services.

Fallow could not be classified using the available data due to its variable land cover characteristics. Fallows were originally defined as fields that have been left to rest for more than two years [23]. Young fallows can look like bare soil or newly harvested fields, while older fallows are similar to shrublands or even have depression-like vegetation cover, due to differences in fertility of the soil, moisture regime and management. This gives fallow a wide range of spectral signatures, and we had to exclude fallow as a social-ecological patch in the maps. 11 percent of the village landscape was classified as fallow fields in the study by Sinare and colleagues [23], making it the third most common social-ecological patch. In other types of ecosystem services assessments this could be problematic, as fallows help restore soil fertility through accumulation of nutrients and organic material. However, of the provisioning ecosystem services in focus in this study, the total generation of services across the landscape is still well estimated, as the set of ecosystem services from fallows can be represented by the combination of shrubland, fields and bare soil. During the interviews, we were also told that fallow practices are decreasing due to increased population pressure, making this a patch of decreasing importance for provisioning ecosystem services.

### Distribution of livelihood benefits across the landscape

**Fig 6:**
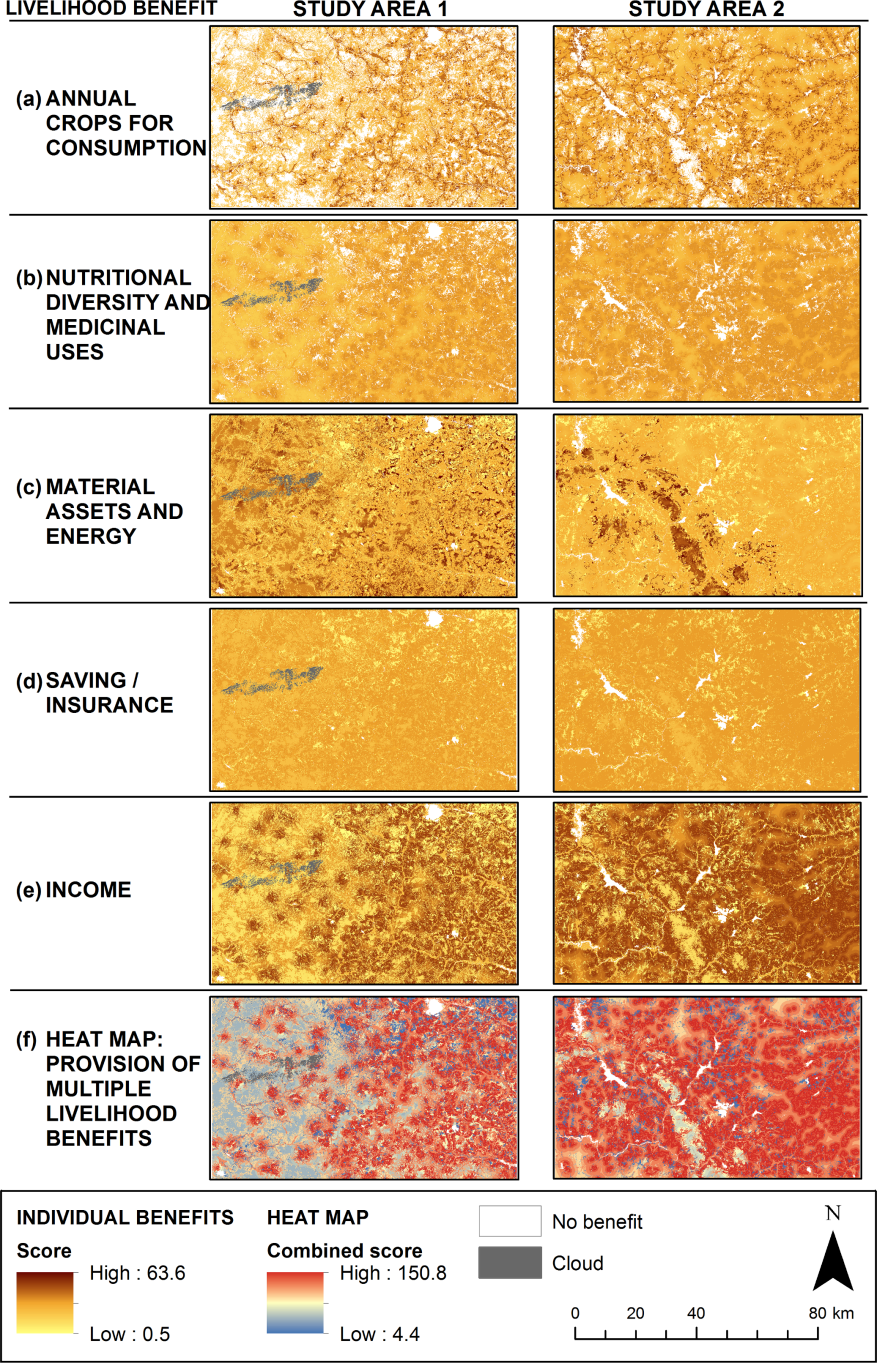
The spatial distribution of livelihood benefits. Maps of the distribution of the five livelihood benefits (a-e) in study area 1 and study area 2 were generated using the social-ecological patch maps and the weighted livelihood benefit scores. The heat maps (f) were generated by adding all separate livelihood benefit maps into one composite map. The heat maps show the provision of multiple livelihood benefits, with high scores suggesting highly multifunctional parts of the landscape.

We generated maps showing the five livelihood benefits by combining the produced social-ecological patch maps and the weighted livelihood benefit scores (Fig 1; section 3.4). The five different livelihood benefits have different distribution patterns across the landscapes in the study areas (Fig 6). Annual crops has the sharpest division between areas with high benefit and no benefit (Fig 6A). Savings/insurance has the most homogenous distribution across the landscape (Fig 6D), as all social-ecological patches provide resources to livestock, which mainly has the benefit of saving or insurance to be used when crops fail or when larger financial resources are needed. This is important as it shows that the whole landscape contributes to building resilience to food insecurity when crops fail. Income has a different distribution pattern as compared to annual crops (Fig 6E). This can be explained by the fact that only parts of annual crops contribute to income. Cereals are not sold, while parts of legumes such as groundnuts and cowpeas are sold and contribute to income. Wild foods from trees and shrubs are also sold to some extent [23]. Nutritional diversity and medicinal uses come from across the landscape, with hotspots around houses (Fig 6B). Material assets and energy come from areas where most of the other benefits are low (Fig 6C). This is because this benefit depends on woodcutting, while other benefits require that woody vegetation is maintained to a high degree.

Heat-maps (Fig 6F) show the overall generation of benefits in different parts of the landscape. Under the current way of managing the landscape, areas with higher value (red) have a more multifunctional use. For example, comparing Fig 6F with Fig 5, areas where homesteads and fields dominate, particularly fields relatively close to homesteads, have high values. This is because fields are used not only for crop production but also to harvest products from trees and for pasture after harvest. The heat-maps indicate that more populated areas generate more benefits from ecosystem services. This is due to the fact that we focused on the local user perspective, and both generation and use of benefits from ecosystem services are included in the maps. Therefore, large areas of e.g. inaccessible shrubland get a lower value per unit area.

## Discussion

### Applicability of method for extrapolating ecosystem service assessments

We have developed a method that, with high accuracy, can map the composition of social-ecological patches in 70–92 percent of the studied landscape. We have also found that the sets of provisioning ecosystem services generated in each patch are valid at the provincial scale. The maps thus give a good overview of the landscape composition in terms of social-ecological patches with local relevance, as well as knowledge of the sets of ecosystem services and livelihood benefits generated at provincial scale in Burkina Faso. The pixel-to-pixel accuracy (producer’s and user’s accuracy, see S2 Table) of the map is lower than the relative accuracy, making it less useful as a detailed map that could be used for e.g. municipal spatial planning purposes. This is important to remember so that maps are used in appropriate contexts, for example meaning that possible future attempts at assessing absolute, rather than relative, values for the landscape provision of different ecosystem services would be less reliable (for a supporting discussion on the hybrid classification method, see S3 Text). After the analysis in this study was finalized, the European Space Agency launched the Sentinel missions, including open access, 10 meter resolution data suitable for vegetation mapping [61]. Developing this method by including the Sentinel dataset in the analysis would likely increase the accuracy of the maps.

As reviewed by Martínez-Harms and colleagues and Malinga and colleagues [6,7], use of secondary data still dominates ecosystem services assessments at the landscape scale, and the few studies that have used primary data for ecosystem services assessments at this scale have only addressed individual services. Recently, further steps have been taken in the development of assessment methods by using focus group discussions and participatory mapping to map multiple ecosystem services in areas with low data availability in Colombia, Nepal and Zanzibar, respectively, connecting ecosystem services to specific places [14,16,17]. However, they did not tie the services to particular landscape units, or analyze the livelihood benefits that these units provide. Instead, they related ecosystem services to particular places, which meant that they are only valid at the study location and not possible to extrapolate.

Thus, our study makes an important contribution to the ecosystem services literature by, identifying social-ecological patches in the landscape based on a local use perspective and explicitly attributing sets of ecosystems services and livelihood benefits to those patches,. This focus on local knowledge and use is a perspective which has been lacking in both ecosystem service assessments [8,62] and policies for natural resources management in development settings in the past.

### Managing the multifunctional land use in the Sahel

Overall, the social-ecological patch and livelihood benefit maps show that the village landscape in the study areas is highly multifunctional, and that the different social-ecological patches contribute to the resilience of the communities in different ways. Our maps point to a number of interesting landscape features that would be worthwhile to explore further. For example, as the livelihood benefit heat maps show (Fig 6F), the total provision of benefits is focused around homesteads and generally high in fields. However, when looking at the spatial distribution of the different livelihood benefits separately (Fig 6), certain trade-offs become evident. Especially, the provision of annual crops for consumption and material assets and energy have an inverse relationship. Also, the benefits of insurance and saving, nutritional diversity, and income have a more even spread across the landscape compared to annual crops for consumption, which is more concentrated and either very high or not provided at all. One reason for this can be that the first group of benefits rely heavily on the practice of integrating shrubs and trees into the field and homestead patches [28]. Trees and shrubs are also fundamental to sustain the ecosystem services used for insurance and saving, which gives resilience to deal with the high variation in crop productivity between wet and dry years [23].

To make informed decisions about livelihood consequences of different investments in the landscape, more detailed knowledge about how the different livelihood benefits are distributed among different user groups in the villages is needed. Previous research has shown that there can be unforeseen trade-offs when an intervention to increase the provision of an ecosystem service is implemented, due to the fact that different user groups benefit to varying degrees from that service [63]. For example, women might benefit more for their incomes from the wild services provided by shrublands than men, meaning that an increase in fields at the expense of shrublands would require that a different source of income is needed for women not to be negatively affected by such an intervention. The existence of these dynamics in the way different groups benefit from the village landscape in the study area needs to be further investigated.

### Future development of the methods

We foresee that the methods for ecosystem services assessments developed here can be used to analyze trade-offs and synergies among ecosystem services when understanding consequences of different land use changes, in the past or for the future. This type of analysis is relevant for assessment of effects on multiple ecosystem services of e.g. expansion of cultivated land, or interventions targeting improved generation of one specific ecosystem service. A potential way to develop our method for these purposes could be to use our generated maps and scores for ecosystem service provision and livelihood benefits as input data into an existing ecosystem service modeling tool such as InVEST [64]. This would increase the relevance of the model output for understanding the connection between ecosystem service supply and human well-being in our particular study area.

A necessary step in order to understand the potential of future interventions in the study areas is to include new social-ecological patches that are increasing, such as small reservoirs and irrigated lands. There was currently only one village with a small reservoir found in each study area, but there is a strong interest in small reservoirs and other water harvesting methods in the region, including dams, boulis (dug hollows in impermeable soil that collect and store rainwater), ponds, half-moons and Zaï pits (dug structures on fields to increase infiltration where crops are planted). Coupling small reservoirs with irrigation schemes would likely show strong effects on nutritional diversity and income [65–67]. The method can be further improved with participatory scoring of the weights describing the effect of distance to homestead and the total area of a social-ecological patch, or other potential aspects affecting the per unit area benefit from a particular social-ecological patch.

It would also be interesting to add analyses of regulating and cultural ecosystem services. However, assigning levels of regulating services to each social-ecological patch is difficult since soil types and vegetation cover is too variable within social-ecological patches to measure differences in indicators e.g. for carbon storage and nutrient circulation [68]. We expect patterns of social-ecological patches in the landscape to play a role for regulating ecosystem services, which can be addressed in a future study. Cultural ecosystem services are not explicitly visible in the map. Spiritual borders, e.g. for sacred groves, cannot be spelled out or drawn on a map due to customary rules [69] which makes this group of ecosystem services less suitable to address with this mapping method. However, a central part of the method is the inclusion of the local user perspective of the landscape, and this use of provisioning ecosystem services cannot easily be separated from the cultural meaning of the landscape and harvested products [70].

## Conclusions

Understanding of landscapes and the benefits these generate for livelihoods requires a combination of relatively in-depth fieldwork at multiple sites to understand the local context and priorities, and remote sensing to be able to assess larger areas. Here, we have presented maps that, with high accuracy, reveal the composition of social-ecological patches in 70–92 percent of the landscape. Based on this we show the spatial distribution of five livelihood benefits in rural northern Burkina Faso. To our knowledge this is the first time that a study has shown the spatial distribution of multiple provisioning ecosystem services across the Sahelian landscape. These types of maps, grounded in local knowledge and extrapolated to regional scale, can be useful both for natural resources management and landscape investments in data scarce regions, and for further research on modeling change in livelihood benefit provision under different landscape changes.

## Supporting information

S1 Fig Schematic illustrating the steps of the method in Sinare and colleagues [23] and in the current study. (TIF)

S2 Fig Photograph of the studied landscape. Taken by first author (KM) in one of the studied villages in study area 1.
(JPG)

S1 Table Specification of raster layers and calculations used for generating livelihood benefit maps. (PDF)

S2 Table Confusion matrix assessing accuracy of social-ecological patch maps. (PDF)

S1 Text Adaption of scores from Sinare and colleagues to current study. (PDF)

S2 Text Additional information about the development of a hybrid classification method. (PDF)

S3 Text Supporting discussion on the developed hybrid classification method. (PDF)

S4 Text Permission from copyright holders for Fig 1. (PDF)

S1 Data Compressed folder containing spatial data layers for study area 1. Included data layers: Depressions shapefile; homesteads shapefile; urban land shapefile; calibration and groundtruthing points shapefile; and social-ecological patch map raster file.
(ZIP)

S2 Data Compressed folder containing spatial data layers for study area 2. Included data layers: Depressions shapefile; homesteads shapefile; urban land shapefile; calibration and groundtruthing points shapefile; and social-ecological patch map raster file.
(ZIP)
