# Supplementary figures and images for: Mapping regional livelihood benefits from local ecosystem services assessments in rural Sahel
Source: PLoS One. 2018 Feb 1;13(2):e0192019. doi: 10.1371/journal.pone.0192019 (PMC5794140; doi:10.1371/journal.pone.0192019)

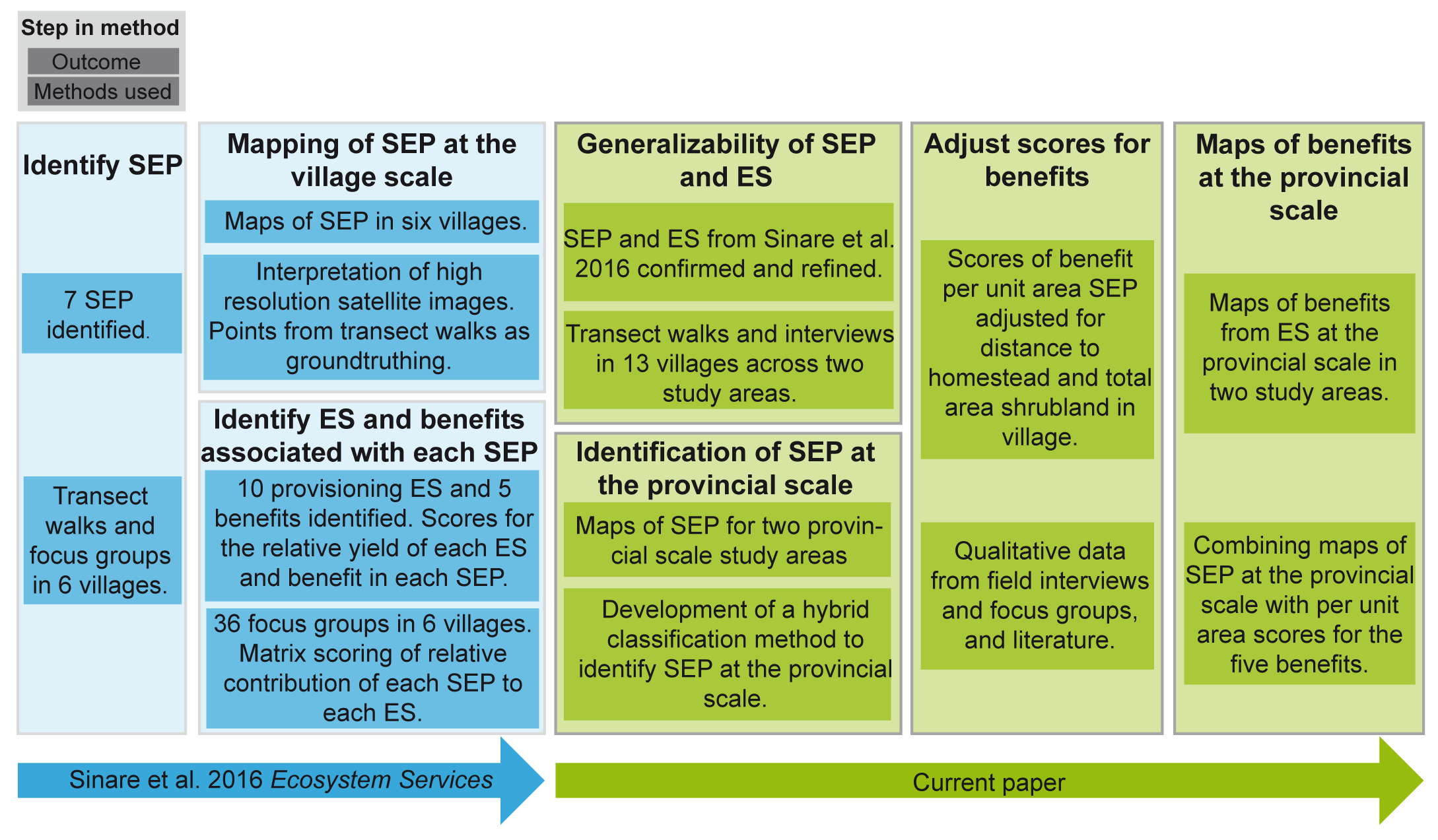

Supplement: S1 Fig — (TIF) [file pone.0192019.s001.tif]

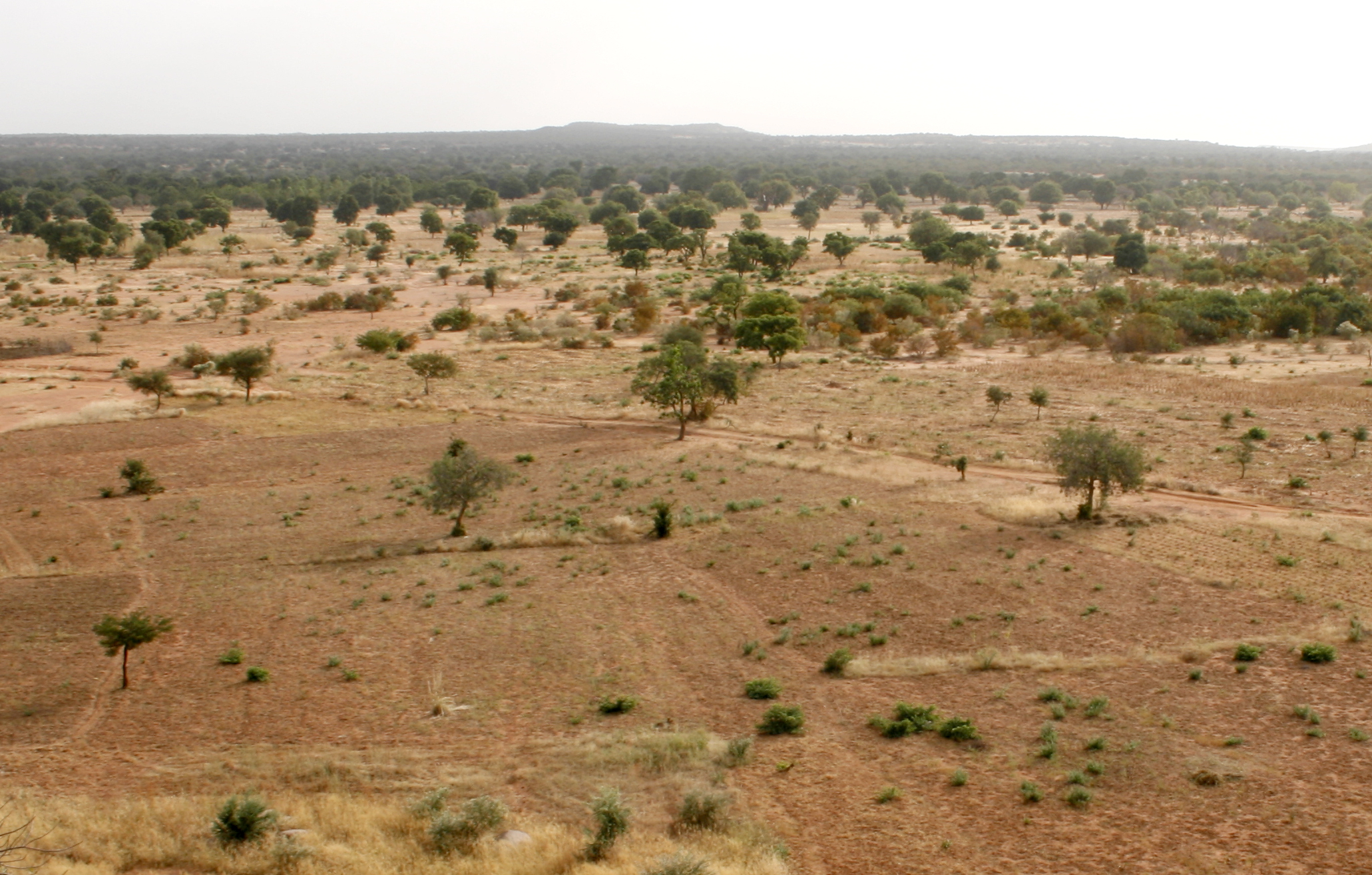

Supplement: S2 Fig — Taken by first author (KM) in one of the studied villages in study area 1. (JPG) [file pone.0192019.s002.JPG]
