# Supplementary material for: Mapping regional livelihood benefits from local ecosystem services assessments in rural Sahel
Source: PLoS One. 2018 Feb 1;13(2):e0192019. doi: 10.1371/journal.pone.0192019 (PMC5794140; doi:10.1371/journal.pone.0192019)
Supplement: S1 Table — (PDF) [file pone.0192019.s003.pdf]

Supporting information for Malmborg et al.

S1 Table. Specification of raster layers and calculations used for generating livelihood benefit maps.

Weight rasters	Homestead buffer zones	0-500 meters from homestead = weight 1.2<br>500-1000 meters from homestead = weight 1.1<br>1000-2000 meters from homestead = weight 1.0<br>2000-3000 meters from homestead = weight 0.9<br>> 3000 meters from homestead = weight 0.8
	Shrubland area categories	0-1 km^2 of shrubland in a village = weight 0.8<br>1-2 km^2 of shrubland in a village = weight 1.2<br>2-3 km^2 of shrubland in a village = weight 1.1<br>3-5 km^2 of shrubland in a village = weight 1.0<br>>5 km^2 of shrubland in a village = weight 0.9
Livelihood benefit maps	Annual crops for consumption	Depression * 62.4 +<br>Homesteads * 75.2 +<br>Fields * 37.6 * Homestead buffer zone weights
	Nutritional diversity and medical uses	Depression * 23.0 +<br>Homesteads * 23.7 +<br>Fields * 21.3 * Homestead buffer zone weights +<br>Shrubland * 18.5 * Homestead buffer zone weights * Shrubland area weights
	Material assets and energy	Depression * 18.1 +<br>Homesteads * 10.4 +<br>Fields * 16.6 * Homestead buffer zone weights +<br>Shrubland * 36.1 * Homestead buffer zone weights * Shrubland area weights +<br>Bare soil * 0.6
	Saving / Insurance	Depression * 21.5 +<br>Homesteads * 18.3 +<br>Fields * 23.7 +<br>Shrubland * 18.3 * Shrubland area weights +<br>Bare soil * 2.7
	Income	Depression * 17.1 +<br>Homesteads * 30.3 +<br>Fields * 31.9 * Homestead buffer zone weights +<br>Shrubland * 9.0 * Homestead buffer zone weights +<br>Bare soil * 0.4
