# Supplementary material for: Mapping regional livelihood benefits from local ecosystem services assessments in rural Sahel
Source: PLoS One. 2018 Feb 1;13(2):e0192019. doi: 10.1371/journal.pone.0192019 (PMC5794140; doi:10.1371/journal.pone.0192019)
Supplement: S2 Table — (PDF) [file pone.0192019.s004.pdf]

Supporting information for Malmborg et al.

S2 Table. Confusion matrix assessing accuracy of social-ecological patch maps[1]

	Study area 1		Study area 2	
**Social-ecological patch**	**Producer's accuracy (%)**	**User's accuracy (%)**	**Producer's accuracy (%)**	**User's accuracy (%)**
Depression	73.8	44.3	80.9	70.3
Homestead	89.4	91.3	92.9	90.7
Field	73.4	54.9	77.2	54.9
Shrubland	38.4	66.3	8.6	18.8
Forest	16.7	33.3	0	0
Bare soil	76.7	41.0	88.0	68.8
Water / Dams	87.5	100	100	100
**Overall accuracy**	59.2	-	68,2	-

[1] Overall accuracy describes the correctness of the classification when all classes are weighed together and compared with the ground truth data. Producer's accuracy shows the likelihood of a groundtruthing point being correctly classified in the map, so called omission errors. User's accuracy describes the likelihood of a classified pixel actually representing what is on the ground, so called commission errors [1]. Using the developed hybrid classification method, depression, homesteads, fields and bare soil could be identified with medium high producer's accuracy (the likelihood of a groundtruthing point being correctly classified in the map). The exclusion of fallow in the classification has consequences for the user's accuracy (the likelihood of a classified pixel actually representing what is on the ground) of all classes, as all fallow groundtruthing points end up in the other classes and are calculated as classification errors. Particularly fields are over-classified in the map due to fallow groundtruthing points classified as fields.

References

1. Congalton RG. A review of assessing the accuracy of classifications of remotely sensed data. Remote Sens Environ. 1991;37: 35–46. doi:10.1016/0034-4257(91)90048-B
