# Supplementary material for: Mapping regional livelihood benefits from local ecosystem services assessments in rural Sahel
Source: PLoS One. 2018 Feb 1;13(2):e0192019. doi: 10.1371/journal.pone.0192019 (PMC5794140; doi:10.1371/journal.pone.0192019)
Supplement: S1 Text — (PDF) [file pone.0192019.s005.pdf]

Supporting information for Malmborg et al.

S1 Text. Adaption of scores from Sinare and colleagues to current paper

The scores used for ecosystem services from social-ecological patches are adapted from Sinare and colleagues [1]. The relative importance of different social-ecological patches for provisioning ecosystem services was assessed by Sinare and colleagues [1] using matrix scoring in focus groups. In each of the six villages, three focus groups with men and three focus groups with women were held (36 focus groups in total) with each group consisting of 5–10 individuals. Photographs with the social-ecological patches and provisioning ecosystem services were used. For each ecosystem service photograph, the focus group was asked to distribute in total 50 beads among the social-ecological patches, representing the relative contribution to particular ecosystem service per unit area of each social-ecological patch. The results were normalised to 100. For example, the relative yields of fruits from different social-ecological patches, normalised to 100, are: depression 29.2, homesteads 14.3, fields 25.0, shrubland 31.5 and bare soil 0. This means that the interviewees assess that the per area yield of fruits are almost the same in shrublands and depressions, slightly lower in fields and only half of that in homesteads. Bare soil generates no fruits.

Three main adaptations to the original work have taken place in this paper. First, since fallow is not part of the present analysis (as this patch could not be identified on provincial scale), the scores for fallows were removed. The scores for the remaining patch categories were adjusted so that their relative importance for each benefit stayed the same, while still normalizing to 100. Second, in the original analysis bare soil got (very low) scores for annual crops, medicines, and shrublands got a (very low) score for annual crops. In discussing this with the villagers in a feedback session of the original work, the villagers argued that this was motivated because these patches had the potential to produce crops if they were to be cultivated (e.g. using soil and water conservation methods to restore bare soils). Since we in this paper look at how the landscape is currently used (not potentially could be used), we decided to assign the score 0 to these social-ecological patches. Finally, in response to discussions during the feedback session, we altered the scores for relative productivity of annual crops so that homesteads was given double the scores as fields, and depression 1.66 times the productivity of fields. The argument from the villagers was that homesteads have in general more intensive management (including more nutrient inputs). Depressions often give

higher yields than fields due to more fertile soils and maintenance of soil moisture during dryspells, but not as high as homesteads.

References

1. Sinare H, Gordon LJ, Enfors Kautsky E. Assessment of ecosystem services and benefits in village landscapes – A case study from Burkina Faso. Ecosyst Serv. Elsevier; 2016;21: 141–152. doi:10.1016/j.ecoser.2016.08.004
