# Supplementary material for: Mapping regional livelihood benefits from local ecosystem services assessments in rural Sahel
Source: PLoS One. 2018 Feb 1;13(2):e0192019. doi: 10.1371/journal.pone.0192019 (PMC5794140; doi:10.1371/journal.pone.0192019)
Supplement: S2 Text — (PDF) [file pone.0192019.s006.pdf]

Supporting information for Malmborg et al.

S2 Text. Additional information about the development of a hybrid classification method

The main data layer for the developed hybrid classification method came from the Landsat 8 OLI sensor [1]. The Landsat 8 OLI sensor contains a refined spectral range in the near infrared band, creating better performance when mapping vegetation [2]. We used the following data layers from or prepared from the dry and rainy season Landsat 8 OLI scenes in the hybrid classification: (i) the red, near infrared and mid-infrared bands (bands 4, 5 and 7); (ii) Normalized difference vegetation index (NDVI) [3,4]; (iii) Tasseled Cap Transformation (TCT), an index that compresses multispectral Landsat data into the three bands brightness, greenness and wetness, to improve accuracy when separating differences in soil moisture, and between brown vegetation and soil [5,6]. Digital elevation models have been shown to improve accuracy of land cover classifications, especially in areas with low vegetation cover [7]. Therefore, two additional data layers were produced in ArcMap [8] based on the ASTER GDEM [9]: (iv) a flow accumulation layer that can be used as a topographical proxy for the likelihood of a pixel getting high water flows, and (v) a slope layer, since steep slopes tend to be shrublands in the study areas (based on field observations).

For two social-ecological patches, depression and homesteads, it was necessary to develop additional data layers. Depression, forest and shrubland all have high values in data layers that indicate vegetation cover. However, depressions are seasonally flooded land, creating a distinct river-like pattern in the images in contrast to the more randomly occurring patches of forest and shrubland. Therefore the Feature Extraction tool in ENVI [10] was used to separate depressions, using the dry season TCT composite image of the study areas, with the flow accumulation layer as ancillary data. The Feature Extraction tool is used to identify patterns between pixels in object-based image analysis and was not applicable for separating the other classes due to the too coarse resolution of the Landsat images. The depressions could not be delineated in one single feature. The feature layer was therefore imported into ArcMap and the depression polygons merged by using the dry season TCT greenness layer as background for visual interpretation.

Homesteads has the most variable land cover of the social-ecological patches and its most distinguishing feature, the houses in the middle, is generally too small to show in the mid-

resolution Landsat images. However, houses are very easy to spot in high-resolution satellite images. Therefore, visual interpretation in Google Earth with a point file of locations of villages in Burkina Faso [11] as a guide for where houses could be expected to occur, was used to pin placemarks in the middle of homesteads for both study areas. This is a time-consuming process and there is a risk of missing some remote and isolated homesteads, but it is easy and the homestead social-ecological patches could not be separated accurately enough using any other method with the available data. The placemarks were exported into ArcMap and 100 meter buffers were created around them to represent the extent of the homesteads.

GPS points from transect walks were used as calibration points. However, as the number of points was small, particularly for some social-ecological patches, the number of calibration points was increased through: (i) Field notes and visual interpretation of the high-resolution satellite images in Google Earth (for forest, depression, shrubland and homesteads); (ii) Extraction of points from village maps of social-ecological patches [12]. However, these points were not allowed to exceed the number of points gathered in the field for each patch, in order for the calibration statistics not to get skewed in favor of the region in the study area where the villages studied by Sinare and colleagues [12] were located. All GPS points were randomly divided into two categories, groundtruthing and calibration points. As far as possible, true ground truth points (points collected during fieldwork) were favored for the first category, and the number of points for groundtruthing was set to at least 30, except for forest in both study areas and bare soil and fallow in the second study area, where the sample was not big enough.

The calibration points were used to calculate mean and standard deviation values for all classes, and the M statistic was used to identify in which layers each class could be separated. The M statistic is a measure that uses the mean and standard deviation of the calibration data of two classes to assess to what degree they are spectrally separable [13,14]. Using the M statistics, as well as results from previous mappings regarding which land covers are generally separable in which data layers [6,7,15], the social-ecological patches were grouped and a decision tree created manually. The parameters for every decision node was set at either one or two standard deviations from the mean of either of the two classes or groups of classes that were to be separated. The decision of which value to set as the separation between classes was partly based on experience from the field. For example, when shurbland and forest were separated, the separating value was set so that the shrubland class would be favored since shurblands are more common in this landscape than forests.

The manually created decision tree was fed into the decision tree tool in ENVI [10] and the resulting classification was visually checked against the calibration points and field observations, then adjusted and reclassified in an iterative process repeated until no more improvements were deemed possible with the available data. The final decision trees are shown in S3 Fig 1.

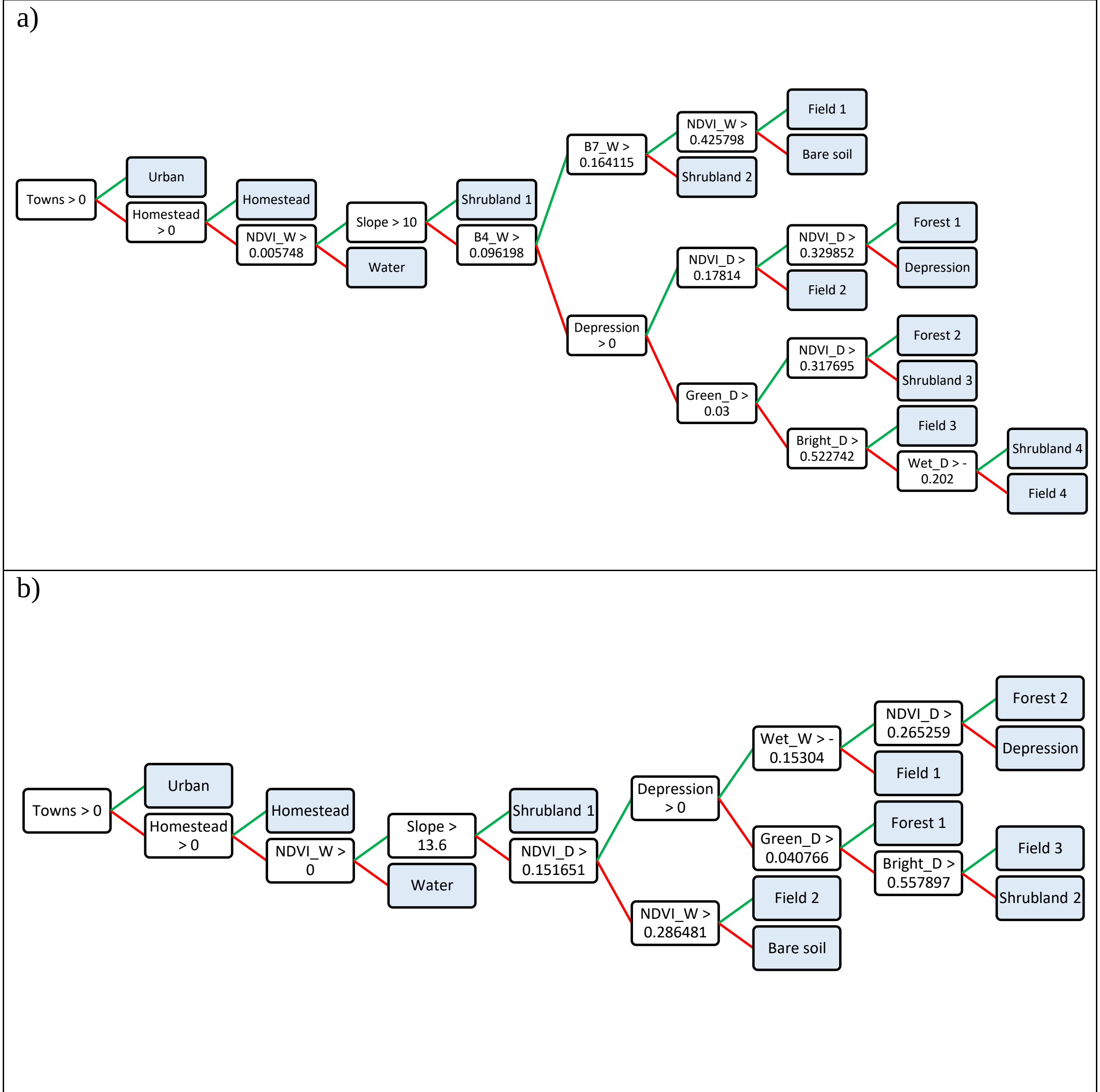

S3 Fig 1. Decision trees developed for study area 1 (a) and study area 2 (b). Green line means yes, red line means no. Legend to abbreviations:

Homestead - layer of homesteads created through visual interpretation in Google Earth and buffering in ArcMap;
Towns - layer of urban areas created through visual interpretation in Google Earth;
Depression - layer of depressions created from a Tasseled Cap Transformation (TCT) of the dry season Landsat 8 OLI scene using feature extraction in ENVI and visual interpretation in ArcMap;
Slope - slope layer calculated with the Slope tool in ArcMap from the ASTER GDEM;
Flow_acc - flow accumulation layer calculated with the Fill, Flow direction and Flow accumulation tools in ArcMap from the ASTER GDEM;
NDVI_D - Normalized Difference Vegetation Index of the dry season Landsat 8 OLI scene;
NDVI_W - Normalized Difference Vegetation Index of the rainy season Landsat 8 OLI scene;
Bright_D/Green_D/Wet_D - The brightness/greenness/wetness layers of the TCT of the dry season Landsat 8 OLI scene, coefficients from [6];
Bright_W/Green_W/Wet_W – brightness/greenness/wetness layers of the TCT of the rainy season Landsat 8 OLI scene, coefficients from [6];
B4_W/B5_W/B7_W - band 4/5/7 of the rainy season Landsat 8 OLI scene.

The accuracy of the produced maps was assessed using groundtruthing points collected during fieldwork. Two versions of the accuracy assessment were produced: one confusion matrix from a pixel-based map comparison with the actual groundtruthing points [16], and one where a 15 meter buffer was created around the points and the accuracy was judged based on if the buffer overlapped with a pixel assigned to the right class. The second version of the accuracy assessment was created as a way to adjust for possible small location inaccuracies of the groundtruthing points from the GPS device, which has a 10 meter GPS accuracy [17], or location inaccuracy in the Landsat 8 OLI scene pixels, as well as for the possibility of a groundtruthing point ending up on the edge of a pixel, giving it a mixed spectral signal. This second type of accuracy assessment is to account for positional errors and, instead of assessing the exactness of the maps, it gives an indication of the relative accuracy of the classification, that is, how well the map captures the composition of the landscape.

References

1. USGS. NASA Landsat Program, Landsat 8 OLI, 2014. In: U.S. Geological Survey Earth Explorer [Internet]. 2015 [cited 19 Jan 2015]. Available: http://earthexplorer.usgs.gov/

2. Jia K, Wei X, Gu X, Yao Y, Xie X, Li B. Land cover classification using Landsat 8 Operational Land Imager data in Beijing, China. Geocarto Int. 2014;29: 941–951. doi:10.1080/10106049.2014.894586

3. Myneni RB, Hall FG, Sellers PJ, Marshak AL. Interpretation of spectral vegetation indexes. IEEE Trans Geosci Remote Sens. 1995;33: 481–486. doi:10.1109/36.377948

4. Mbow C, Fensholt R, Rasmussen K, Diop D. Can vegetation productivity be derived from greenness in a semi-arid environment? Evidence from ground-based measurements. J Arid Environ. Elsevier Ltd; 2013;97: 56–65. doi:10.1016/j.jaridenv.2013.05.011

5. Dymond CC, Mladenoff DJ, Radeloff VC. Phenological differences in Tasseled Cap indices improve deciduous forest classification. Remote Sens Environ. 2002;80: 460–472. doi:10.1016/S0034-4257(01)00324-8

6. Baig MHA, Zhang L, Shuai T, Tong Q. Derivation of a tasselled cap transformation based on Landsat 8 at-satellite reflectance. Remote Sens Lett. 2014;5: 423–431. doi:10.1080/01431160110106113

7. Keshtkar HR, Azarnivand H, Arzani H, Alavipanah SK, Mellati F. Land Cover Classification Using IRS-1D Data and a Decision Tree Classifier. 2013;17: 137–146.

8. ESRI. ArcGIS Desktop: Release 10.3. Redlands, CA: Environmental Systems Research Institute; 2013.

9. NASA LP DAAC. ASTER GDEM V2, 2011. In: A product of METI and NASA. NASA EOSDIS Land Processes DAAC, USGS Earth Resources Observation and Science (EROS) Center, Sioux Falls, South Dakota [Internet]. 2011 [cited 26 Aug 2014]. Available: http://reverb.echo.nasa.gov/

10. EVIS. ENVI version 5.1. Boulder, Colorado: Exelis Visual Information Solutions;

2013.

11. IGBF. Bf_loc.shp. Used with special permission from Institut Geographique du Burkina Faso; 2014.

12. Sinare H, Gordon LJ, Enfors Kautsky E. Assessment of ecosystem services and benefits in village landscapes – A case study from Burkina Faso. Ecosyst Serv. Elsevier; 2016;21: 141–152. doi:10.1016/j.ecoser.2016.08.004

13. Kaufman YJ, Remer L a. Detection of forests using mid-IR reflectance: an application for aerosol studies. IEEE Trans Geosci Remote Sens. 1994;32: 672–683. doi:10.1109/36.297984

14. Sesnie SE, Finegan B, Gessler PE, Thessler S, Bendana ZR, Smith AMS. The multispectral separability of Costa Rican rainforest types with support vector machines and Random Forest decision trees. Int J Remote Sens. 2010;31: 2885–2909. doi:10.1080/01431160903140803

15. Sharma R, Ghosh a., Joshi PK. Decision tree approach for classification of remotely sensed satellite data using open source support. J Earth Syst Sci. 2013;122: 1237–1247.

16. Stehman S V. Sampling designs for accuracy assessment of land cover. Int J Remote Sens. 2009;30: 5243–5272. doi:10.1080/01431160903131000

17. Garmin. GPSMAP 62-serien användarhandbok. Olathe, Kansas: Garmin International Inc.; 2011.
