# Supplementary material for: Mapping regional livelihood benefits from local ecosystem services assessments in rural Sahel
Source: PLoS One. 2018 Feb 1;13(2):e0192019. doi: 10.1371/journal.pone.0192019 (PMC5794140; doi:10.1371/journal.pone.0192019)
Supplement: S3 Text — (PDF) [file pone.0192019.s007.pdf]

Supporting information for Malmborg et al.

S3 Text. Supporting discussion on the developed hybrid classification method

We have developed a method that with high accuracy can map the composition of social-ecological patches in 70-92 percent (relative accuracy) of the studied landscape. The pixel-to-pixel accuracy (producer's and user's accuracy) of the map is lower than the relative accuracy, making it less useful as a detailed map. This is important to remember so that maps are used in appropriate contexts, for example meaning that possible future attempts at assessing absolute, rather than relative, values for the landscape provision of different ecosystem services would be less reliable. There are also limits to the replicability of the method, as it is not fully automatic, even though the M statistic [1,2] also was used to guide the selection of data layers and parameters used for separating different classes. The method was developed in an iterative process where decisions by the developer and knowledge about local conditions were crucial.

Higher resolution data would have increased the accuracies of the maps, making object-based classification [3] possible, which potentially could make classification of depressions and homesteads more automatic. Object-based classification might also have been successful for classification of fields. The landscape in both study areas is generally flat, with a few exceptions. Even small topographical differences make a big difference for water availability and soil moisture. Therefore, a higher-resolution, better quality digital elevation model could have increased accuracy, especially for the shrubland, depression and bare soil social-ecological patches. It is also possible that reducing the size of the study areas could have increased the accuracy. However, this would also have decreased the efficiency of the method, since it would have meant that more mappings would have been needed for the same scale of coverage. Therefore, depending on what the maps are going to be used for, the right balance between accuracy and coverage needs to be set. The higher number of villages used for groundtruthing in study area 1 (eight villages) compared to study area 2 (five villages) did not seem to make a difference for the accuracies of the most common social-ecological patches (field, homestead and depression). However, for the rarer social-ecological patches (forest and shrubland), the lower number of groundtruthing points appears to have decreased the accuracies considerably. For example, shrubland had a 50.0 percent relative accuracy in study area 1, while the same measure for study area 2 is 11.4 percent. This suggests that if

these rarer social-ecological patches are important for the intended use of the maps, a higher number of study villages is recommended. However, due to the low sample size with regards to studied villages, we cannot be sure that the difference in accuracies is due to the difference in number of villages in each study area. The composition of the landscape in study area 1 and 2 is different, with study area 2 being more homogenous with less woody vegetation (based on field observations and from studying high-resolution satellite images in Google Earth of the study areas). This difference in landscape composition could also affect the extent to which the identification of different social-ecological patches was successful.

A limitation of the method is the time consuming initial phase, where social-ecological patches need to be identified and mapped in detail to create both classes and calibration data to be used in the hybrid method [4]. In other contexts with new patches, new indicators for the decision tree must also be developed and added to the hybrid method. In case the map coverage needed requires the use of several Landsat scenes, adjustments also need to be made to the decision tree, preferably even using separate calibration and groundtruthing data for separate scenes. What all of these limitations suggest, is that there is a trade-off between creating a classification with high social relevance and the efficacy in map production. We argue that the strength of the method developed in this paper is that it allows for the inclusion of rich local and socially relevant information, while at the same time being generalizable to a scale that is relevant for decision making with mostly high accuracy through a relatively simple classification procedure.

References

1. Kaufman YJ, Remer L a. Detection of forests using mid-IR reflectance: an application for aerosol studies. IEEE Trans Geosci Remote Sens. 1994;32: 672–683. doi:10.1109/36.297984
2. Sesnie SE, Finegan B, Gessler PE, Thessler S, Bendana ZR, Smith AMS. The multispectral separability of Costa Rican rainforest types with support vector machines and Random Forest decision trees. Int J Remote Sens. 2010;31: 2885–2909. doi:10.1080/01431160903140803
3. Blaschke T. Object based image analysis for remote sensing. ISPRS J Photogramm

Remote Sens. Elsevier B.V.; 2010;65: 2–16. doi:10.1016/j.isprsjprs.2009.06.004

4. Sinare H, Gordon LJ, Enfors Kautsky E. Assessment of ecosystem services and benefits in village landscapes – A case study from Burkina Faso. Ecosyst Serv. Elsevier; 2016;21: 141–152. doi:10.1016/j.ecoser.2016.08.004
