# Supplementary material for: Mapping regional livelihood benefits from local ecosystem services assessments in rural Sahel
Source: PLoS One. 2018 Feb 1;13(2):e0192019. doi: 10.1371/journal.pone.0192019 (PMC5794140; doi:10.1371/journal.pone.0192019)
Supplement: S4 Text — (PDF) [file pone.0192019.s008.pdf]

Stockholm January 9, 2017

We hereby accept the open-access journal PLOS ONE to publish Figure 1 in the manuscript PONE-D-16-37790 *Mapping Regional Livelihood Benefits from Local Ecosystem Services Assessments in Rural Sahel* under the Creative Commons Attribution License (CCAL) CC BY 4.0 (http://creativecommons.org/licenses/by/4.0/). The figure is a derivative of Figures 3a and 4c in our article Sinare, H., Gordon, L. J, Enfors Kautsky, E. 2016. Assessment of ecosystem services and benefits in village landscapes – A case study from Burkina Faso. *Ecosystem Services* 21: 141-152, to which we are copyright holders.

Hanna Sinare

Line Gordon

Elin Enfors Kautsky
