# Supplementary material for: Mapping regional livelihood benefits from local ecosystem services assessments in rural Sahel
Source: PLoS One. 2018 Feb 1;13(2):e0192019. doi: 10.1371/journal.pone.0192019 (PMC5794140; doi:10.1371/journal.pone.0192019)
Supplement: S2 Data — Included data layers: Depressions shapefile; homesteads shapefile; urban land shapefile; calibration and groundtruthing points shapefile; and social-ecological patch map raster file. (ZIP) [file pone.0192019.s010.zip › SA2/SA2_METADATA.pdf]

METADATA S8 Data.zip

File name	Description
SA2_Depression.shp	Shapefile with depression polygons in study area 2.<br><br>**File type:** Shapefile<br><br>**Description/Key attribute table**<br>*SEP*: Social-ecological patch<br><br>**Credits**: Katja Malmborg
SA2_GT_C.shp	Groundtruthing and calibration points for study area 2<br><br>**File type**: Shapefile<br><br>**Description/Key attribute table**<br>*Point_ID*: ID number for points<br>*Lat*: Latitude<br>*Long*: Longitude<br>*Village*: Village in which point is located. 'Other' signifies points where name of village was not known.<br>*Type1*: Groundtruthing point (GT) OR calibration point (C)<br>*Type2*: 1. Point collected with GPS device during fieldwork; 2. Points created by using field notes and located using Google Earth; 3. Points created from data in Sinare et al. (2016); 4. Points located through visual interpretation in Google Earth.<br>*SEP*: Social-ecological patch (Classes: Bare_soil; Depression; Field; Forest; Homestead; Shrubland; Water; Urban)<br><br>**Credits**: Katja Malmborg
SA2_HS.shp	Shapefile with homesteads in study area 2.<br><br>**File type**: Shapefile<br><br>**Description/Key attribute table**:<br>*SEP*: Social-ecological patch<br><br>**Credits**: Katja Malmborg
SA2_SEP_map.tif	Raster file of social-ecological patches in study area 2.<br><br>**File type**: Geo TIFF<br><br>**Description/Key attribute table**:<br>*SEP:* Social-ecological patch (Classes: Bare_soil; Depression; Field; Forest; Homestead; Shrubland; Water; Urban)<br><br>**Credits**: Katja Malmborg

SA2_Urban.shp	Shapefile with urban land in study area 2.<br><br>**File type**: Shapefile<br><br>**Description/Key attribute table**:<br>*SEP*: Social-ecological patch<br>*Name*: Name of town<br><br>**Credits**: Katja Malmborg
